# Influence of Virtual Reality Devices on Pain and Anxiety in Patients Undergoing Cystoscopy Performed under Local Anaesthesia

**DOI:** 10.3390/jpm11111214

**Published:** 2021-11-16

**Authors:** Mateusz Łuczak, Łukasz Nowak, Joanna Chorbińska, Katarzyna Galik, Paweł Kiełb, Jan Łaszkiewicz, Andrzej Tukiendorf, Katarzyna Kościelska-Kasprzak, Bartosz Małkiewicz, Romuald Zdrojowy, Tomasz Szydełko, Wojciech Krajewski

**Affiliations:** 1 University Center of Excellence in Urology, Department of Urology, Wrocław Medical University, 50-556 Wrocław, Poland; mateusz.luczak@umw.edu.pl (M.Ł.); katarzyna.galik@umw.edu.pl (K.G.); romualdzrojowy@umw.edu.pl (R.Z.); 2 University Center of Excellence in Urology, Department of Minimally Invasive and Robotic Urology, Wrocław Medical University, 50-556 Wrocław, Poland; lukasz.nowak@umw.edu.pl (Ł.N.); joanna.chorbinska@umw.edu.pl (J.C.); pawel.kielb@umw.edu.pl (P.K.); jasieklaszkiewicz@gmail.com (J.Ł.); bartoszmalkiewicz@umw.edu.pl (B.M.); tomaszszydelko@umw.edu.pl (T.S.); 3 Department of Public Health, Wrocław Medical University, 51-618 Wrocław, Poland; andrzej.tukiendorf@umw.edu.pl; 4 Department of Nephrology and Transplantation Medicine, Faculty of Medicine, Wroclaw Medical University, 50-367 Wrocław, Poland; katarzyna.koscielska-kasprzak@umw.edu.pl

**Keywords:** virtual reality, cystoscopy, bladder cancer, pain, fear

## Abstract

Background: Bladder cancer is one of the most common malignancies. Its diagnosis is based on transurethral cystoscopy. Virtual reality (VR) is a three-dimensional world generated through the projection of images, the emission of sounds and other stimuli. VR has been proven to be a very effective “distractor” and, thus, a useful tool in managing pain. The aim of this study was to determine whether the use of VR sets is technically feasible during the cystoscopy and whether the use of VR devices would reduce the degree of ailments associated with the procedure; Methods: The study prospectively included both men and women who qualified for rigid cystoscopy due to both primary and follow-up diagnostics. The study group underwent rigid cystoscopy with the VR set and the control group underwent the procedure without the VR set. Patients enrolled in both groups were subjected to blood pressure, heart rate and saturation measurements before, during and after the procedure. Additionally, the patients were asked to describe the severity of fear, pain sensations and nausea associated with the procedure. Non-verbal pain manifestations were assessed using the adult adjusted Faces, Legs, Activity, Cry and Consolability (FLACC) scale; Results: The study population included 103 patients (74M/29F; mean age 64.4 years). Pain intensity differed significantly between the groups, reaching lower values in the VR group. In all analyzed subgroups the use of the VR set was associated with higher levels of nausea. The mean FLACC score reached higher values for patients without the VR set. Blood pressure as well as heart rate increased during the procedure and decreased afterwards. The increase in systolic blood pressure and pulse rate was statistically higher in the control group; Conclusions: This study confirmed that cystoscopy is associated with considerable preprocedural fear and severe pain. Blood pressure and heart rate rise significantly during the cystoscopy. VR sets can lower pain perception during cystoscopy, but they may cause moderate nausea.

## 1. Introduction

Bladder cancer (BCa) is one of the most common malignancies. The diagnosis of BCa is mainly based on transurethral cystoscopy (CS) [1]. In the majority of cases, CS is performed under local anaesthesia. Therefore, CS is associated with some ailments [2].

Virtual reality (VR) is defined as a three-dimensional world generated through the projection of images, the emission of sounds and the production of other stimuli. It enables “immersion” in a virtually-created environment [3]. Realistic interactions with VR through goggles, headphones, special gloves and other devices make it possible to manipulate, operate and control virtually generated objects [4].

VR sets were originally developed as military training tools and entertainment devices. However, in recent years, VR has been proven to be a very effective “distractor” and thus, a useful tool in managing pain perception.

The aim of the study was to determine whether the use of VR sets is technically feasible during the transurethral CS performed under local anaesthesia. In addition, we aimed to prove whether the use of VR devices would reduce the degree of ailments associated with the procedure.

## 2. Materials and Methods

The study protocol was approved by the Institutional Bioethics Committee (consent number KB-276/2020 06.05.2020). The study prospectively included both men and women who qualified for rigid cystoscopy due to suspected BCa (primary diagnostics) or due to periodic surveillance after the previous surgical treatment of BCa. Exclusion criteria involved medical contraindications for a VR set (anatomical deformities as well as otolaryngologic, ophthalmologic, neurologic issues), indwelling catheters, CS with intervention, symptomatic urinary tract infection, history of Bacillus Calmette-Guérin (BCG) immunotherapy and inability to cooperate with psychological evaluations. Patients taking medications that could affect their mental states were also disqualified.

The randomization was performed according to previously computer-generated schemes. The patients were not informed which group they were assigned to until the start of the procedure. The first group underwent rigid CS with the VR set consisting of goggles and headphones (VR group), and the second group underwent rigid CS without the VR set (control group).

The content presented by the VR device was selected after a consultation with ophthalmology, neurology, and otolaryngology specialists, as well as with VR professionals.

The procedure details and technical parameters of the device were set and adjusted during the preliminary tests carried out on members of the research team during the simulated procedure, and additionally, during several CS procedures not included in this analysis.

Because of the intellectual limitations resulting from age and comorbidity, we chose an image presenting a nature landscape (the Skogafos waterfall in Iceland). The image was static to the viewing point and dynamic in terms of the animal activity, plant movement and flowing water. When the subject’s head moved, the image shifted, imitating the sensation of “looking around”. The headphones emitted sounds that imitated the naturally-occurring sounds at the Skogafos waterfall (nature sounds). The intensity of the emitted sound was adjusted so that it was well tolerated by the examined patient, but it also significantly limited the perception of the operating theater noises (sounds of electronic devices, tools, conversations) and prevented communication with the staff.

One urologist (M.Ł.) conducted the vast majority of the procedures and measurements. All CSs were performed in the dorsal lithotomy position, using a lubricating gel containing 2% lidocaine without any systemic sedation or analgesia. Rigid 20 Fr cystourethroscopes were used. Patients had the opportunity to observe the CS on the screen.

Patients enrolled in both groups were subjected to detailed clinical observation, including blood pressure (BP), heart rate (HR) and blood oxygen saturation analysis. The measurements were collected in 4–5-min intervals, and the mean values were calculated for the pre-, peri- and postoperative periods. The standard cardiac monitor device was used. Self-perception of fear, pain and nausea were assessed using a numeric rating scale (NRS) ranging from 0 (e.g., free from pain) to 10 points (e.g., unbearable pain). Perception of fear was assessed before the randomization. Pain and nausea perception were assessed a few minutes after the CS completion. The adult-modified Face, Legs, Activity, Cry, Consolability scale (FLACC) was used to assess nonverbal pain manifestations by the assisting nurse, with scores ranging from 0 (no pain) to 10 points (unbearable pain).

Based on the preliminary assessment (data not shown), we calculated that 100 patients were sufficient to achieve desired statistical power. The comparisons were analyzed with the Mann–Whitney and Wilcoxon signed-rank tests. The variations of frequencies were tested using Chi-squared tests. The value of the adjusted *p <* 0.05 was considered statistically significant.

## 3. Results

The study included 29 females and 74 males (mean age: 66.4 years). The basic characteristics of the patients are presented in Table 1. The main study group did not differ significantly in terms of basic parameters.

Table 2 presents the results of the pre-procedural fear, pain perception, nausea and FLACC assessment in all patients included in the study. The results are divided by the main study groups, as well as by gender. The CS result (negative—no tumor/positive—tumor visualized) and CS history (primary/consecutive) did not influence any of the analyzed parameters (data not shown).

Table 3 shows the results of the homeostasis parameters measured at various study timepoints. As the parameters were measured various times during each study period in 4–5-min intervals, the results in the table are presented as the average values of all measurements taken at a given timepoint of the study (pre-, peri- and postprocedural). The results are divided by the main study groups, as well as by gender. The CS result or CS history did not influence any of the analyzed parameters (data not shown).

Table 4 presents the absolute differences between the mean values of each parameter measured at the given study timepoint. The results are divided by the main study groups, as well as by gender.

## 4. Discussion

In this study, we aimed to assess whether the use of the VR set during rigid CS would reduce the discomfort associated with the procedure. We found that the use of VR was feasible and the VR set was generally well tolerated. Only a few patients reported moderate vertigo or nausea. However, none of the CS procedures were interrupted because of patients’ ailments. The patients did not report any additional problems related to the device that were reasons to stop the procedure.

The study confirmed the previous observations that rigid CS is an unpleasant and stressful procedure [2]. The patients presented moderate levels of preprocedural fear. Whereas no patients described their fear as level 0, some patients described their fear as level 8–10. In addition, the fear levels were higher in patients who were undergoing their first CS (data not shown).

In our study, we hypothesized that it would be more suitable to measure fear than anxiety during the short, invasive procedure such as CS. Fear is not widely recognized as a psychopathological symptom. Hence, no previous studies have assessed fear (as an isolated parameter) of CS. However, unlike anxiety, which is considered a diffuse, unfocused, objectless and future-oriented feeling, fear is considered a reaction to a specific, observable danger. Both states, although clearly overlapping, are considerably different. Anxiety is significantly more often associated with muscle tension, vigilance in anticipation of the expected danger, caution (often excessive) and avoidance behaviors. On the other hand, fear is much more often accompanied by a significant intensification of the autonomic stimulation to fight, as well as thoughts of sudden danger (e.g., CS) [5]. In our study, this was confirmed by the preprocedural measurements of the BP and HR. Both values clearly exceeded the norms and typical distribution in the Polish population. When analyzed by gender, the fear level was significantly more pronounced in women. Unfortunately, it was unclear whether the differences resulted from real disparities or from different societal roles of men and women.

In the available literature, several studies have analyzed the issue of pain during cystoscopy. The described methods of pain alleviation include the use of lidocaine lubrication, high pressure of the irrigation solution and additional maneuvers, such as real-time self-visualization, listening to music, the assisting nurse holding the patient’s hand, watching television and using a stress-ball [6,7,8,9,10,11]. In addition, researchers have also studied classic pharmacotherapy [12]. However, the use of VR has not been thoroughly researched. Only one randomized study has studied VR in flexible CS, which included a small sample of patients [13,14]. The authors did not show significant pain reduction in the VR group, likely because a flexible device was used [2]. Another study focused on VR in one patient undergoing transurethral microwave thermotherapy [15].

In this study, the pain levels during CS in the control group did not differ from previous findings. The results clearly show that the pain perception in males was significantly higher than in females. Whereas some men described the pain as level 10 (unbearable), some women reported the pain as severe as level 8. This is contrary to the widespread dogma that female CS is painless.

The pain level associated with VR CS was significantly lower when compared to the control group. The mean difference reached almost 1 unit. However, this difference was due to differences in the male CS. In females, there was no observable difference in pain sensations between the VR and control group.

In addition, patients in the control group showed more pronounced nonverbal pain manifestations as assessed by the FLACC scale. Contrary to the NRS pain measurements, this difference was much more evident in the female population.

The influence of pain and fear on homeostasis parameters has been widely proven [16]. Therefore, in addition to the subjective psychometric assessment, we also analyzed the objective indicators of homeostasis, such as BP, HR and blood oxygen saturation. The change in these objective parameters results from the stimulation of the sympathetic nervous system by pain or various psychological factors. This stimulation is believed to be the result of an evolutionary mechanism of “escape” from a dangerous situation, or the preparation for “fight” or “defense”. Therefore, these factors are an objective measure of the actual level of physiological “stress” [5].

In this study population, despite the elevated preprocedural homeostasis parameters, significant increases were observed in both BP and HR during cystoscopy. However, the increase was notably less intense in the VR group. These observations were consistent for both genders and might suggest that using VR alleviated some pain or at least efficiently distracted the patients from the negative experience. Shortly after the procedure, the parameters returned to the values recorded before CS in the majority of patients. Again, the change was less severe in patients that received the VR set. Contrary to the fear assessment, the CS result and the number of previous CSs had no influence on the BP and HR (data not shown).

Throughout the procedure, the blood oxygen saturation levels were normal in the vast majority of patients. In addition, no significant changes were observed during CS. Although the stress and pain experienced before and during the cystoscopy may have influenced the patients’ breathing patterns, the difference was minor, and the resulting changes did not exceed physiological values.

This study faces some limitations. First, it was conducted in one center and in a relatively small population. Second, the VR equipment showed only passive, stationary images without any active tasks (e.g., games). Due to the BCa population characteristics and its limitations, we assumed that active interactions within VR might be too burdensome for the patients. Third, the homeostasis parameters used in the study are not specific to CS. In addition, the FLACC scale was originally developed to analyze the pediatric population. We used an adult-adjusted FLACC scale, as there is no widely used tool to describe nonverbal pain manifestations in adults. Finally, psychometric questionnaires are subjective, and the answers are based on each patient’s comprehension of the questions. Psychometric questionnaires can be influenced by a variety of factors including the disease itself, and are not specific to CS.

## 5. Conclusions

This study confirmed that CS is unpleasant for patients, especially for men. CS is associated with considerable preprocedural fear, as well as severe pain during the procedure. BP and HR rise significantly during the CS. Moreover, patients presented various nonverbal manifestations of intense pain. Using VR equipment during CS is feasible, but it may cause moderate nausea and vertigo. VR sets can lower pain perception, especially in males.

## Figures and Tables

**Table 1:** Patients’ basic characteristics.

	All Patients (*n* = 103)	VR Group (*n* = 52; 50.5%)	Control Group (*n* = 51; 49.5%)	*p*
Gender (M/F)	74/29 (71.8/28.2%)	38/14 (73.1/26.9%)	36/15 (70.6/29.4%)	0.779
Age (mean; SD)	66.4; 11.3	65.6; 11.4	67.3; 11.1	0.396
Cystoscopy (primary/consecutive)	48/55 (46.6/53.4%)	25/27 (48.1/51.9%)	23/28 (45.1/54.9%)	0.761
Cystoscopy result (negative/positive)	73/30 (70.9/29.1%)	38/14 (73.1/26.9%)	35/16 (68.6/31.4%)	0.619

Abbreviations: M, male; F, female; SD, standard deviation; VR, virtual reality.

**Table 2:** Analyzed ailments associated with cystoscopy in various study groups. The results are presented as mean, standard deviation and range.

	All Patients (*n* = 103)	VR Group (*n* = 52; 50.5%)	Control Group (*n* = 51; 49.5%)	*p*	Males VR (*n* = 38; 36.9%)	Males without VR (*n* = 36; 34.9%)	*p*	Females VR (*n* = 14; 13.6%)	Females without VR (*n* = 15; 14.6%)	*p*
Preprocedural fear	3.8 ± 2.3 [1÷10]	3.8 ± 2.5 [1÷10]	3.8 ± 2.2 [1÷9]	0.651	3.3 ± 2.1 [1÷10]	3.4 ± 2.1 [1÷9]	0.800	4.9 ± 2.9 [1÷10]	4.8 ± 2.1 [3÷8]	0.949
Pain perception	4.7 ± 2.4 [1÷10]	4.3 ± 2.2 [1÷10]	5.1 ± 2.6 [1÷10]	0.049 *	4.6 ± 2.3 [1÷10]	5.6 ± 2.3 [1÷10]	0.003 *	3.6 ± 1.8 [1÷8]	3.4 ± 2.1 [1÷8]	0.354
Nausea	1.4 ± 1.1 [1÷7]	1.8 ± 1.4 [1÷7]	1.1 ± 0.3 [1÷2]	0.001 *	1.7 ± 1.1 [1÷6]	1.1 ± 0.3 [1÷2]	0.005 *	2.0 ± 2.0 [1÷7]	1.0 ± 0 [1÷1]	0.000 *
FLACC	1.6 ± 1.8 [0÷7]	1.3 ± 1.7 [0÷6]	2.0 ± 1.9 [0÷7]	0.026 *	1.4 ± 1.6 [0÷6]	1.7 ± 1.7 [0÷6]	0.287	0.7 ± 1.6 [0÷6]	2.6 ± 2.3 [0÷7]	0.023 *

* *p <* 0.05. Abbreviations: VR, virtual reality; FLACC, Face, Legs, Activity, Cry, Consolability scale.

**Table 3:** Homeostasis parameters at various study timepoints.

	All Patients		VR Group		Control Group		*p*	Males VR		Males without VR		*p*	Females VR		Females without VR		*p*
	Mean	SD	Mean	SD	Mean	SD		Mean	SD	Mean	SD		Mean	SD	Mean	SD	
Preprocedural systolic BP	137.4	23.4	137.2	25.1	137.7	21.5	0.900	139.4	26.8	140.1	22.2	0.965	131.1	18.2	131.7	18.1	0.747
Preprocedural diastolic BP	81.2	13.7	83.4	14.9	79.0	11.9	0.273	83.9	17.0	77.8	13.0	0.223	82.0	6.5	81.8	8.0	0.949
Periprocedural systolic BP	157.2	23.3	154.0	26.6	160.5	18.8	0.136	156.1	28.3	160.8	18.2	0.289	148.2	20.2	159.5	20.3	0.252
Periprocedural diastolic BP	88.8	14.0	89.4	16.3	88.2	11.0	0.673	90.3	17.1	88.3	10.9	0.555	87.2	13.6	88.1	11.3	0.949
Postprocedural systolic BP	144.1	19.1	143.7	19.1	144.5	19.1	0.843	144.8	18.3	145.4	18.8	0.957	140.6	20.8	142.3	19.7	0.813
Postprocedural diastolic BP	83.4	11.7	86.0	11.6	80.9	11.2	0.051	86.7	12.7	81.5	10.7	0.083	84.1	7.5	79.3	12.1	0.270
Preprocedural HR	77.7	13.7	79.0	13.4	75.7	13.9	0.120	78.5	13.7	76.8	14.0	0.384	80.3	12.3	72.4	13.2	0.134
Periprocedural HR	82.9	15.9	82.2	14.2	83.7	17.5	0.934	81.6	14.2	86.8	18.3	0.417	83.8	14.2	76.1	12.5	0.158
Postprocedural HR	76.0	13.7	78.3	13.0	73.6	14.1	0.048 *	78.5	13.2	75.7	14.8	0.167	77.9	12.1	68.7	10.9	0.057
Preprocedural saturation	96.9	1.9	97.0	2.0	96.8	1.9	0.785	96.7	1.8	96.6	1.9	0.546	97.6	2.2	97.3	1.5	0.642
Periprocedural saturation	97.2	1.9	96.7	1.9	97.8	1.8	0.811	96.4	1.7	97.6	1.8	0.781	97.2	2.2	98.2	1.7	0.551
Postprocedural saturation	97.1	2.2	97.2	2.2	97.0	2.2	0.931	97.0	2.3	96.6	2.3	0.399	97.8	1.6	97.8	1.5	0.797

* *p <* 0.05. Abbreviations: BP, blood pressure; HR, heart rate; SD, standard deviation; VR, virtual reality.

**Table 4:** The absolute differences between the mean values of each parameter measured at the given study timepoint.

	VR Group Pre- vs. Peri-	Control Group Pre- vs. Peri-	*p*	VR Group Peri- vs. Post-	Control Group Peri- vs. Post-	*p*
Systolic BP	16.8	22.8	0.029 *	10.3	15.9	0.006 *
Diastolic BP	6.0	9.2	0.256	3.4	7.4	0.086
HR	3.2	8.1	0.009 *	3.9	10.0	0.000 *
Saturation	0.3	0.5	0.152	0.6	0.8	0.321
	**Females VR Pre** **- vs.** **Peri-**	**Males VR Pre- vs. Peri-**	***p***	**Females VR Peri** **- vs.** **Post-**	**Males VR Peri- vs. Post-**	***p***
Systolic BP	17.1	16.7	0.773	7.5	11.3	0.509
Diastolic BP	5.2	6.3	0.556	3.0	3.6	0.570
HR	3.5	3.1	0.802	5.9	3.2	0.157
Saturation	0.2	0.3	0.311	0.3	0.7	0.109
	**Females without VR Pre- vs. Peri-**	**Males without VR Pre- vs. Peri-**	***p***	**Females without VR Peri- vs. Post-**	**Males without VR Peri- vs. Post-**	***p***
Systolic BP	27.8	20.7	0.075	17.2	15.4	0.756
Diastolic BP	10.4	6.3	0.316	8.9	6.7	0.463
HR	3.8	9.9	0.049 *	7.4	10.0	0.218
Saturation	0.3	0.6	0.398	0.7	0.9	0.198

* *p <* 0.05. Abbreviations: BP, blood pressure; HR, heart rate; Pre-, preoperative measurement; Peri-, perioperative measurement; Post-, postoperative measurement; VR, virtual reality.

## Data Availability

The dataset used and/or analyzed during the current study is available from the corresponding author upon reasonable request.
